# Sex-Related Effects in the Superhydrophobic Properties of Damselfly Wings in Young and Old *Calopteryx splendens*

**DOI:** 10.1371/journal.pone.0088627

**Published:** 2014-02-10

**Authors:** Katja Kuitunen, Alexander Kovalev, Stanislav N. Gorb

**Affiliations:** 1 Department of Biological and Environmental Science, University of Jyväskylä, Jyväskylä, Finland; 2 Department of Functional Morphology and Biomechanics, Zoological Institute, University of Kiel, Kiel, Germany; Uppsala University, Sweden

## Abstract

Numerous sex-related morphological adaptations are connected to reproductive behavior in animals. For example, females of some insect species can submerge during oviposition, which may lead to sex-related adaptations in the hydrophobicity (water-repellency) due to specialization of certain morphological structures. On the other hand, ageing can cause changes in hydrophobicity of the surface, because the morphological structures can wear with age. Here, we investigated sex-and age-related differences in wing hydrophobicity and in morphology (spine density, wax cover characteristics, size of females' pseudopterostigma) potentially related to hydrophobicity of *Calopteryx splendens* damselflies. Hydrophobicity was measured with two methods, by measuring the contact angle (CA) between a wing and water droplet, and by dipping a wing into water and measuring forces needed to submerge, withdraw, and pull-out a wing from water. We found that *C. splendens* wings are superhydrophobic, having mean CAs of 161°. The only sex and age related difference in the hydrophobicity measurements was that young females had stronger amplitude of force fluctuations during withdrawal of wings from water than young males. This suggests that young females may form less uniform air pockets on their wings while submerged. From the morphological structures measured here, the only sex related finding was that old females had denser spine cover than young females in their wing veins. The difference may be explained by better survival of females with denser spine cover. The most important morphological character that predicted superhydrophobicity was the prevalence of long wax rods on wing veins. In addition, female pseudopterostigma area (a trait present only in females) was negatively related to pull-out force, suggesting that large pseudopterostigmas might help females to emerge from water following oviposition. The subtle sex-related differences in hydrophobicity could be explained by the fact that both sexes must resist rain, and males are occasionally in contact with water.

## Introduction

Selection pressures due to sex specific behavior can cause adaptations in morphological structures. Many sex differences in behavior and morphology are related to reproduction. For example, females give birth or lay eggs, and therefore have specific adaptations corresponding to this part of their life cycle. In some semiaquatic insects, females lay their eggs while partly or totally submerged in water [1] [2], and therefore may be expected to have greater water repellency than males as a result of natural selection. Support for this kind of adaptation is found in the damselfly *Calopteryx cornelia*, in which females submerge during oviposition [1]. Females of this species are able to survive under water longer than males, suggesting that there could be sex-specific adaptations related to submerging. In addition, there are some indications that the wings of female damselflies might have denser bristle cover than males, which could mediate differences in the hydrophobicity of the wing surface between the sexes [1].

A surface is superhydrophobic if a water droplet forms a contact angle (CA) greater than 150° with the surface and the water droplet easily rolls on the surface [3] [4]. Superhydrophobicity (CA>150°) is explained by the Cassie-Baxter state of wetting: solid-liquid contact is reduced as water contacts the tops of cuticle protrusions and leaves air pockets in gaps between protrusions [5]. In contrast, in the Wenzel state of wetting the gaps between protrusions are filled with water, and in such a case the surface is often hydrophobic but not superhydrophobic (90°<CA<150°) [6]. The Cassie-Baxter state and the Wenzel state are extremes of the same continuum and transitional states are likely to occur [3] [4] [7] [8] [9] [10] [11]. Superhydrophobicity is often caused by hierarchical micro- and nanoscale structures [12] [13] [14] [15] [16] [17] [18] [19]. For example, microscale hairs (microsetae) and nanoscale pattering on such hairs are responsible for the ability of water striders to move along water surfaces without the danger of drowning [13] [14] [17]. Hierarchical morphology is thought to increase the stability of superhydrophobicity [10]. Artificial hierarchical structures with combinations of micro- and macroscopical levels have stronger hydrophobicity than structures with only either level alone [9]. Structures of different size also have different contributions to the effect of superhydrophobicity, e.g. larger level structures repel larger water droplets while smaller structures resist micro-sized droplets [15] [16]. The pockets of air retained by morphological structures can diminish with time, because air diffuses into the surrounding water and leads to the reduction of superhydrophobicity [4] [20]. Sometimes superhydrophobic surface structures can also include hydrophilic structures to increase the stability of air pockets responsible for superhydrophobicity of an organism [21].

Superhydrophobicity of wings and other parts of terrestrial and semi-aquatic insects can provide many benefits. For example, superhydrophobic surfaces reduce the likelihood of being trapped by water, provide resistance to the wetting effect of condensed dew, they increase exposure to the self-cleaning properties of rolling water droplets, and they inhibit wing weight increase which could impair flying [9] [10] [15] [16] [18] [22] [23]. One important benefit of superhydrophobicity is that it can help an insect to retain air around the body and wings as “external lungs” when submerged. Use of an air pocket or bubble (plastron) while submerged occurs in insects and arachnids, and it enables access to oxygen when they are submerged. In addition to retained air, dissolved oxygen can diffuse to the air pocket from surrounding water [1] [2] [20] [24]. An air pocket may also contribute to the drag reduction due to the reduction of the solid-liquid contact [8] [21] and help generate adhesion to submerged surfaces [25]. Superhydrophobic structures can also have additional benefits which are not related to contact with water. For example, superhydrophobic surfaces with microcrystalline wax coverage may have anti-reflective characteristics, thereby decreasing visibility to predators [23] [26], and reducing heat loadings [28].

Superhydrophobicity may decline as an insect ages, since structures generating the effect can wear off or become damaged over time, or the chemistry of the structures may change (see e.g. [19]). Nanostructures are especially susceptible to damage when mechanically challenged [3]. If structures responsible for superhydrophobicity are damaged, an insect may risk entrapment by water surface tension or drown. Accumulation of water droplets or dirt may also increase an organism's weight thus increasing exposure to predators [3]. The effect of age on the nanoscale wax coverage has been previously studied in *Calopteryx splendens* and *Calopteryx virgo* damselfly males [27]. Surprisingly, in these species the condition of wax layer on abdomen is better in older than younger males. It cannot be excluded, however, that this difference is a result of better survival of males with good quality wax coverage [27].

*Calopteryx* damselflies are semiaquatic insects, in which larval development takes place in water, and adults are aerial predators which perform their reproductive activities at streams and rivers. Similar to other odonates (i.e. damselflies and dragonflies), representatives of the genus *Calopteryx* have dense layer of crystalline wax that covers almost the whole body including wings [27] [29] [30]. The wax cover increases hydrophobicity in odonates [31]. In addition, there are other cuticle protuberances, such as bristles or spines at the microscale, which could have an effect on superhydrophobicity in Odonata [1]. *Calopteryx* damselfly females are more often in contact with water than males, because they lay their eggs into aquatic plants while partially or completely submerged [1]. Males come in a close contact with water less frequently and for shorter durations. They can be pushed to water by conspecific males when they are fighting over patches of aquatic vegetation for territories used as oviposition sites by females. When ovipositing females are partially submerged, males can also try to intitiate the first stages of reproduction by clasping the females prothorax [32] [33], and may also land onto the water surface during some stages of their courting display [34]. Because of such behavioural differences between males and females, we hypothesize that females are under stronger selection to resist water than males, and especially well adapted to submerge. Short exposures to water, for example under impact of rain drops, have likely not contributed to differences in selection pressures between the sexes. Taking all that in to account, sex-related adaptations for water resistance could be possible in *Calopteryx* damselflies.

In this study we investigated the water resistance of wings of *Calopteryx splendens* males and females, belonging to two age groups. We concentrated on wings, as the potential for interplay with water is great due to the large size of wings [15] [16] [23], and because wing condition is critically important for adult survival. First, we investigated whether there are sex or age differences in the hydrophobicity of wings by measuring contact angles between a wing surface and water droplet, and by measuring force needed to dip a wing into and out from water. Immediately following each submersion with a dry wing the wing dipping phase was repeated with wet wing, allowing us to investigate possible sex- and age-related differences between these two conditions. Second, we measured micro- and nanoscale characteristics (density of spines on wing veins, characteristics of wax crystal coverage) in both males and females of each age group, because sex differences in these morphological characters could reflect possible adaptations to water resistance. Third, we investigated whether superhydrophobicity of the wing surface correlated with micro- and nanostructural characteristics. The correlation between the area of the pseudopterostigma and the hydrophobicity measurements was also explored. Pseudopterostigma exists only in females, and their microstructure differs from that of normal wing (see Results section), thus having the potential to contribute to sex differences in water resistance.

## Methods

### Ethics statement

No licences or permission were needed for the study, because the study species is not protected in Finland, and the study area was not protected either. In Finland, there is a public right to access to land by every person irrespective of land ownership.

### Animals

*C. splendens* females and males (*n* = 43) were collected from River Niemenjoki (Central Finland N: 62°15′; E: 26°19′) in 2007. Specimens were subdivided in two groups: young (1–2 days after reaching maturity) and old (at least 10 days after reaching maturity). The age of individuals were obtained by mark-recapture (see [27] for further details). Sample sizes were such that there were 13 young and 11 old females, and 12 young and 7 old males. The average lifespan of the mature phase of *C. splendens* males is approximately 4–6 days [35] [36], meaning that the 10 day old individuals used in this study were twice as old as an average individual. The damselfly samples were collected in small containers in the field and brought to laboratory, where they were immediately euthanized by freezing for approximately 30 min at −20°C. Dried specimens were stored at room temperature until measurement in 2010. Wing length was measured from the left hind wing of each individual with digital calliper to nearest 0.01 mm.

### Contact angle (CA) measurements

By measuring the contact angle between a surface and a water droplet, it is possible to estimate the hydrophobicity or hydrophilicity of the surface. CA measurements were carried out with the Contact Angle System OCA 20 (DataPhysics Instruments, Filderstadt, Germany). We made measurements at the middle section of each hind wing of each *C. splendens* individual, i.e. one hind wing was measured on the dorsal and the other on the ventral side. The wing was attached with double sided tape to a glass slide and a 1 µl droplet of distilled water was placed on the wing with a thin needle. A needle-in method was applied [37], because the wing surface was so hydrophobic that it was not possible to withdraw the needle and obtain a stable droplet for a contact angle measurement. Although other tests for superhydrophobicity (i.e. low roll-off angle or high drop mobility [3]) were not directly tested here, it was apparent that the roll-off angle must be very low, because the droplet was not stable at even the almost flat horizontal position of the wing. The angle between the droplet and wing surface was measured using SCA20 software (v.3.12.11, DataPhysics Instruments). Measurements were repeated five times per wing side.

In addition to above procedure, cryo-scanning electron microscopy (cyro-SEM; see section “SEM and morphological analyses”) was performed on a small subset of wings (n = 3) to investigate the shape individual droplets attached on wings, whether droplets attached on wing membranes and/or on wing veins, and whether wax damage was related to droplet location. This was done by spraying a wing with distilled water and then freezing a wing with liquid oxygen (−147°C), after which the wing was immediately examined in SEM.

### Force measurements (buoyancy and capillary force)

Wing tips were dipped into water to measure forces resisting wing submersion and affecting withdrawal of a wing from water. To perform these measurements, the base of each front wing was attached using double sided adhesive tape to the tip of a force tranducer (FORT25, World Precision Instruments, Sarasota, FL, USA) mounted on a motorized micromanipulator (DC3001R, World Precision Instruments, Sarasota, FL, USA). Such an experimental design allowed us to measure forces acting on the wing. Only wings in good condition (no cracks or bending underwater) were used (n = 29, including 4 young and 9 old females, 11 young and 5 old males). The wing tip was submerged in 20 ml of distilled water at the speed of 0.2 mm per second. On average, 9.9±0.7 mm (mean ± SD) of the wing tip was submerged. The average time of submergence was 19.0±13.9 s, after which the wing was withdrawn from the water. Immediately after the first dipping, the procedure was repeated for a wet wing measurement. Forces were measured using a MP100 data acquisition system and recorded with AcqKnowledge 3.7.0 – software (Biopac Systems, Inc.) with a polling frequency of 200 Hz.

**Figure 1:**
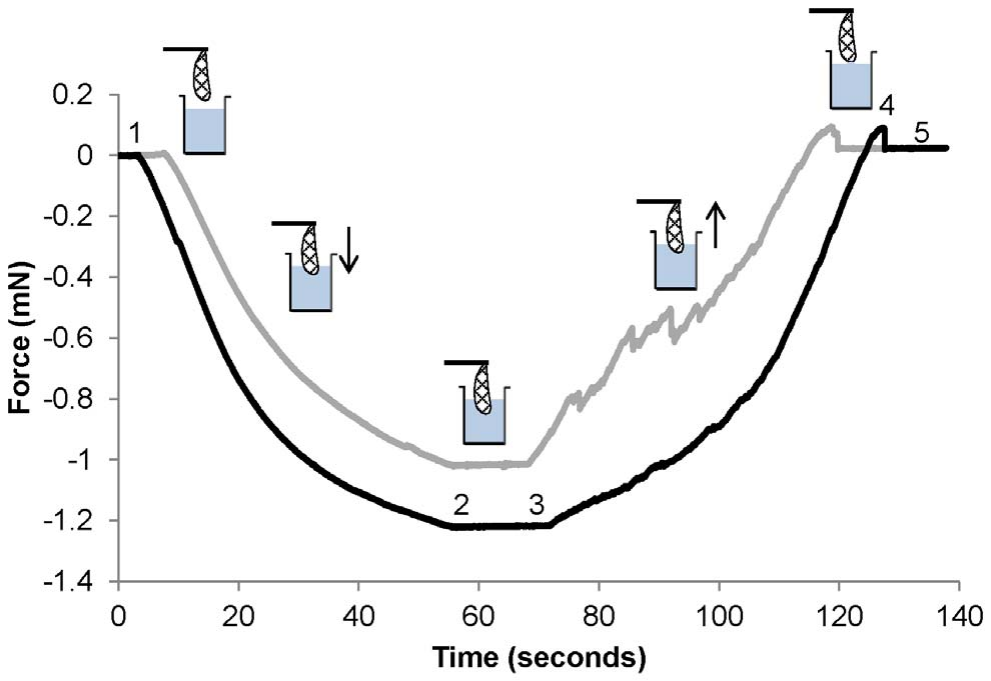
Two force curves obtained during wing submersion experiments including both the dipping phase (1–2) and the withdrawal phase (3–4). The black curve shows a typical force curve with a minor degree of fluctuation amplitude during withdrawal. The grey curve is from an individual which had more force oscillation during withdrawal. Different steps of trials are marked with numbers to the black curve: 1) starting point of dipping the wing into the water (background level of the force before dipping), 2) stop of dipping, 3) start of wing withdrawal, 4) peak value, indicating the pull-out force needed to detach the wing from the water when the force is compared to step 5), the wing in the air (background level of the force after dipping). Negative values indicate the buoyant force, and positive values indicate surface tension forces. Time (s) is counted from the beginning of each trial. Note that here the force is not normalized to the wing area.

Several parameters were determined from the force curves obtained. First, the force needed to submerge the wing tip, i.e. the buoyant force, was calculated as a difference between the force value before dipping, and a force value after the tip of a wing was submerged (steps #1 and #2 on Fig. 1). Here we assume as buoyant force both buoyant and surface tension forces. The buoyant force values were normalized by the dipped area of a wing. The estimation of the dipped area of a wing included two steps: First, the length of dipped part of the wing from wing tip to its base was calculated based on the time-force displacement curves from the dipping trials (the dipping velocity was constant, see above). Second, the wings were scanned (scanner: Ricoh Aficio MP C3500, 300 dpi) and the area of dipped part of a wing were measured with software (see below) based on the length estimated during the previous step.

During the withdrawal phase of a trial the buoyant force sometimes strongly fluctuated (the curve between steps #3 and #4 in Fig. 1) whereas during the dipping phase of a trial (the curve between steps #1 and #2 in Fig. 1) the buoyant force increased homogeneously. The fluctuation amplitude in the withdrawal curves was estimated by fitting the withdrawal curves with third order polynomial (i.e. cubic curves), and estimating standard deviations from the residuals. The third order polynomial fitted well to the withdrawal curves, *R* ^2^ = 0.99±0.01 (mean ± SD). Finally, pull-out force, which is needed to pull the wing tip off the water, was estimated as a difference between the positive peak value of the force measurement curve and the background level immediately after the wing was out of the water (steps #4 and #5 in Fig. 1). The pull-out force is the force which is needed to overcome the capillary force. The pull-out force was not divided by area of a wing submerged, because this force corresponds to only the cross section of the very tip of the wing that has the last contact with water.

### SEM and morphological analyses

Scanning electron microscope (SEM) photographs were taken from the central portion of the hind wings of each individual (n = 43) to determine micro- and nanostructure of *C. splendens* wings. One wing was measured from dorsal and the other wing from ventral side. Small pieces of hind wings were excised and mounted on aluminum holders using double-sided sticky conductive tape. The preparations were sputter-coated with gold–palladium (thickness 10 nm), and examined in a SEM Hitachi S-4800 at 3 kV. Photographs were taken at five positions of the wing membrane and wing veins at a magnification of ×5000, and five additional photographs were taken of the wing membrane at a magnification of ×15000. One out of five wing membrane measurements was taken from the wing nodus but the four others and all wing vein photos were taken from randomly selected places. We avoided photographing areas where there was damage caused by handling of the wings by the experimentalist.

**Figure 2:**
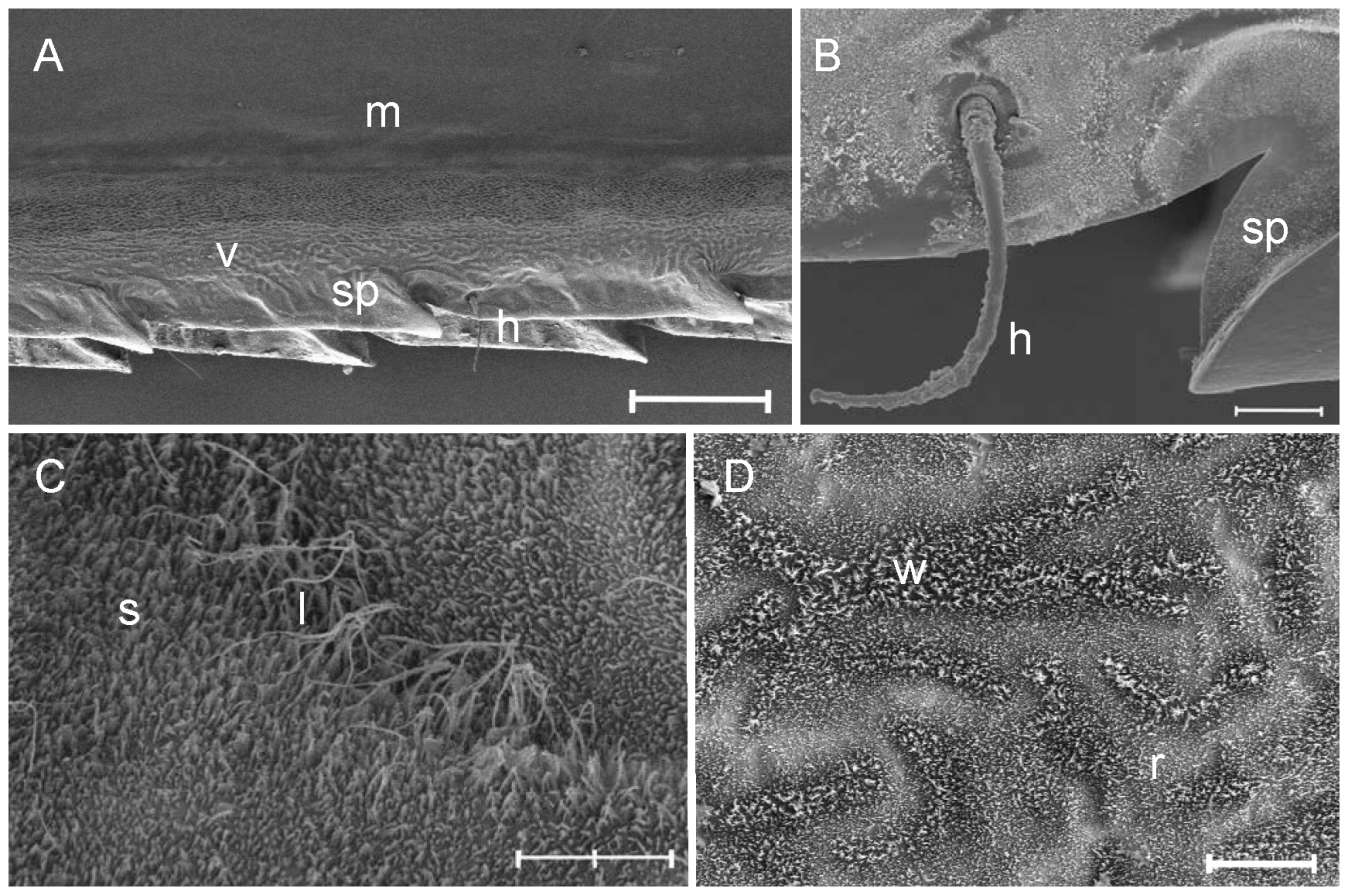
SEM photographs of micro- and nanostructure characteristics of *C. splendens* wing cuticle. A) Costal vein from nodus region. Two rows of spines, one from dorsal (uppermost) and the other from ventral side of a wing. Spine tips are oriented toward the direction of the wing tip. B) There are tiny hairs next to spine tips on lateral sides of wings; C) Long, filamentous wax rods on a wing vein; D) Details of female pseudopterostigma. Symbols: h  =  hair, l  =  long wax rods, m  =  wing membrane, r  =  ridge pattern of wing membrane; s  =  short wax rods, sp  =  spine; v  =  wing vein, w  =  wax rods. Scale bars: A: 60 µm, B: 6 µm C: 600 nm, D: 4 µm.

Spine densities on wing veins were measured along the costal vein, from the nodus to the direction of wing tip (Fig. 2 A, B). The costal vein was photographed at magnification of ×100, and the distance between the first and the eighth spines was measured. In the analyses, the average distance between two consecutive spines (i.e. the distance between the first and the eight spines divided by seven) was used as a variable indicating spine density, because it was not always possible to measure distance of all eight spines. The spine density did not correlate with wing length (i.e. size of an individual) (Pearson correlation, *r* = −0.12, *N* = 43, *P* = 0.454). Thus, the wing length was not taken into account in further statistical analyses concerning the spine density.

**Figure 3:**
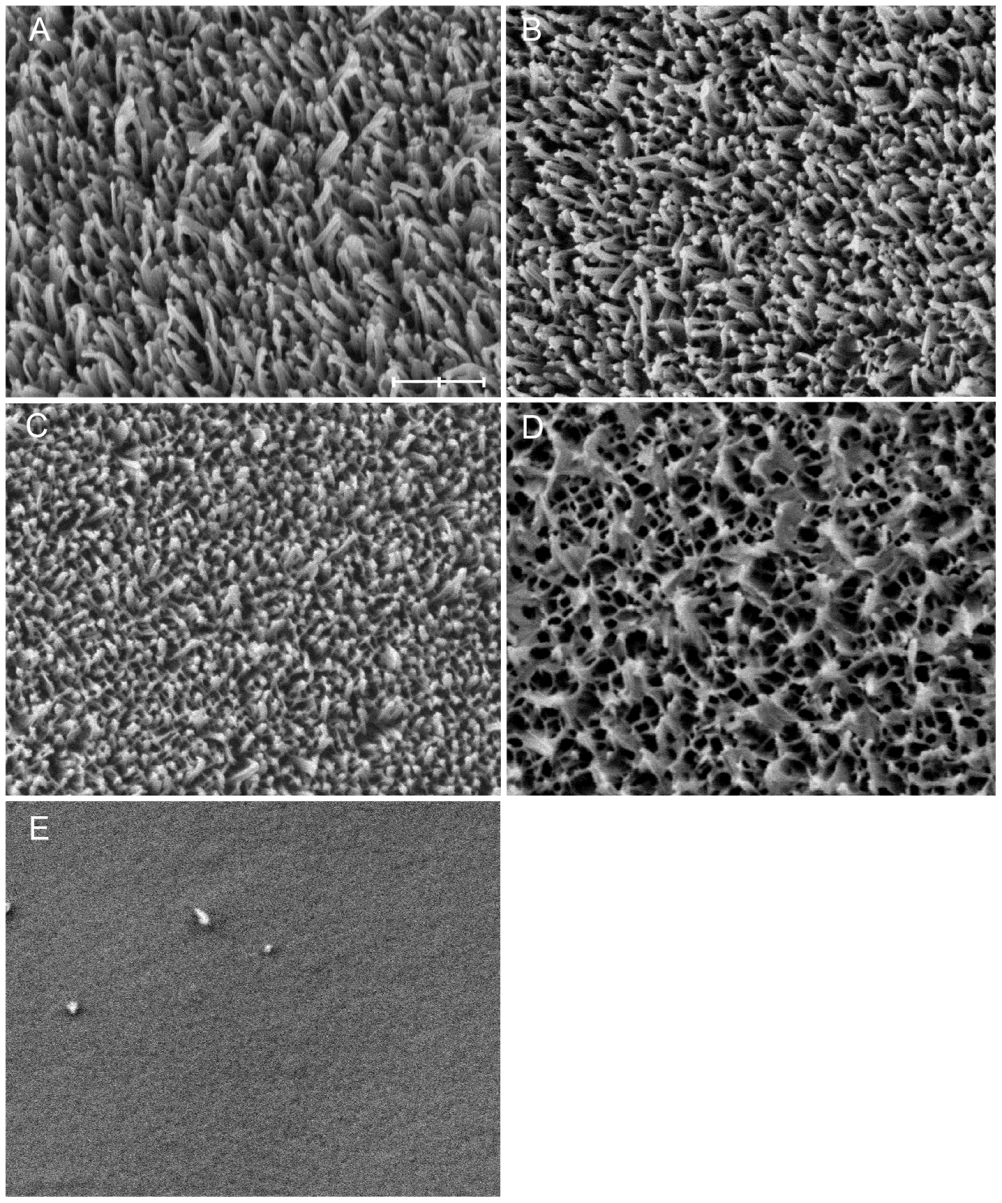
The damage of rod-like wax coverage was divided into five classes: A) intact (class 1), B) wax rods slightly joined together (class 2), C) wax rods are strongly joined and the coverage looks network-like (class 3), D) wax crystals are joined together, forming lumps (class 4), E) wax rods are lost (class 5). The most samples belonged to the classes two or three. Scale bar = 600 nm.

The properties of wax coverage were measured in three ways: First, a proportion of wax coverage which was lost from or smeared on the wing surface was measured from the ×5000 magnification photographs by dividing the area of lost or smeared wax by the total photographed area (50 µm^2^). This was done for the wing membrane and wing vein photographs separately, leading to four measures: 1. proportion of lost wax from wing membrane, 2. proportion of smeared wax on wing membrane, 3. proportion of lost wax from wing vein and 4. proportion of smeared wax on wing vein. Second, ×15000 magnification photos of wing membranes were examined and damage was classified on a scale ranging from 1 to 5, in which 1 described intact wax crystals and 5 described a cuticle surface in which only traces of wax were left (see Fig. 3 for more detailed classification). SEM photographs with contaminated cuticle surfaces were omitted from classification. Finally, to determine how common long, filamentous, wax rods are on wing veins (Fig. 2C), the proportion of ×5000 magnification vein photos including filamentous wax were counted. Hereafter, this variable is called as the prevalence of wing veins covered with long wax rods.

Only female *C. splendens* have a pseudopterostigma at their wing tips. Because of this sex difference, pseudopterostigmas may have some role in repelling water as only ovipositing females completely submerge. To test this hypothesis, we measured the pseudopterostigma area from either front wing for every female in the dipping trials (*N* = 13). This was done by scanning the wings with a computer scanner (Ricoh Aficio MP C3500, 300 dpi) and measuring the area. All area measurements from images were obtained with the aid of Sigma Scan Pro - software (version 5.0.0).

### Statistical analyses

CAs and most morphological traits (excluding wing lengths and the areas of pseudopterostigma) were measured several times per each individual. For such traits, means for each individual were used in the statistical analyses.

A 2-way ANOVA was used to investigate the effects of sex and age on CA values. Three separate repeated measures ANOVAs were employed to investigate the effects of sex, age, and trial on the three force variables investigated (buoyant force, amplitude of force fluctuations during withdrawal, and pull-out force).

Micro- and nanostructure comparisons between sex and age groups were done with MANOVA, after which separate 2-way ANOVAs for each dependent variable were performed. To reduce the number of dependent variables, a principal component analysis (PCA) by using covariance matrices was applied to the four wax characteristics (smeared wax on wing membrane and veins, lost wax from wing membrane and wing veins) before the analyses; these principal components were used instead of original values. Two principal components (PC) were obtained: PC1, representing the combination of the smeared wax in wing veins and the lost wax in wing veins (Pearson correlation: *r* = 0.97, *N* = 43, *P*<0.001, and *r* = 0.40, *N* = 43, *P* = 0.009, respectively; explains 28.4% of the total variance), and PC2, describing only the lost wax in wing veins (*r* = 0.92, *N* = 43, *P*<0.001; explains 22.5% of the total variance). Thus, the dependent variables in the MANOVA model were the density of spines in wing veins, the damage class of wax, the proportion of wing veins including long wax rods, PC1, and PC2. The independent variables in the MANOVA model were age, sex, and the interaction between age and sex. Finally, age group differences in pseudopterostigma areas of females were compared with independent samples t-test.

Linear multiple regression analyses were conducted to determine whether contact angle and force measurements were related to micro and nanostructures (spine density, the proportion of wing veins with long wax rods, PC1, and PC2). Damage class was left out from regression analyses to avoid collinearity problems; the variable was closely correlated with the proportion of wing veins containing long wax rods (Pearson correlation, *r* = −0.36, *N* = 43, *P* = 0.016). Since the first contact of wing with water is biologically the most relevant one, the dependent variables only included force measurements from the first dipping trial when wings were initially dry. Non-significant explanatory variables were excluded in a stepwise manner. Separate models were generated for each measurement of hydrophobicity, i.e. CA, buoyant force, fluctuations in amplitude of the buoyant force during withdrawal of a wing, and pull-out force. In addition, separate correlations were performed to investigate a possible relationship between the area of pseudopterostigma and each measure of hydrophobicity (i.e. CA and all three force measures from the first dipping trial).

The fluctuation amplitude of the buoyant force during withdrawal of a wing from the water and pull-out force were log_10_-transformed to fulfill the assumptions of the statistical tests. The pull-out force did not fulfill all of the assumptions even after the transformation, but the deviation from the assumptions were less with the log_10_-transformed data (data not shown). All statistical analyses were conducted with SPSS (v.18.0.0), and all reported *P*-values are two tailed.

## Results

### Contact angle measurements

All wings were superhydrophobic with contact angles varying between 156° and 165° (mean ± SD  = 161±2°). There were no sex or age differences nor an interaction between the two variables on the contact angle between water droplets and damselfly wings (2-ANOVA, sex: *F*_1,39_ = 0.75, *P* = 0.393, age group: *F*_1,39_ = 1.89, *P* = 0.177, sex*age group *F*_1,39_ = 0.49, *P* = 0.488).

**Figure 4:**
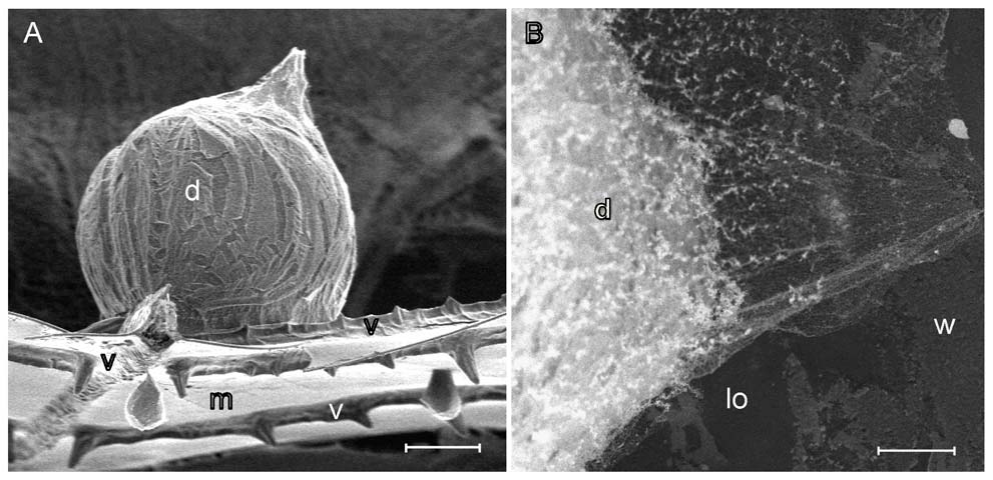
Cryo-SEM of a small water droplet on a wing. A) Lateral view of wing and spherical water droplet; B) Dorsal view of wing and water droplet. In this case, the droplet is attached on a place of membrane from which the wax coverage has been damaged. Symbols: d  =  water droplet, lo  =  lost and damaged wax coverage, m  =  wing membrane, v  =  wing vein, w  =  wax rods. Scale bars, A: 40 µm and B: 10 µm.

Cryo-SEM revealed that water droplets have spherical shape on wing surface (Fig. 4), verifying the superhydrophobic nature of the wing surface. In the wing membrane, the droplets attached to damaged areas, but on wing veins, the droplets were found on both wax damaged areas and on undamaged areas.

### Force measurements

**Figure 5:**
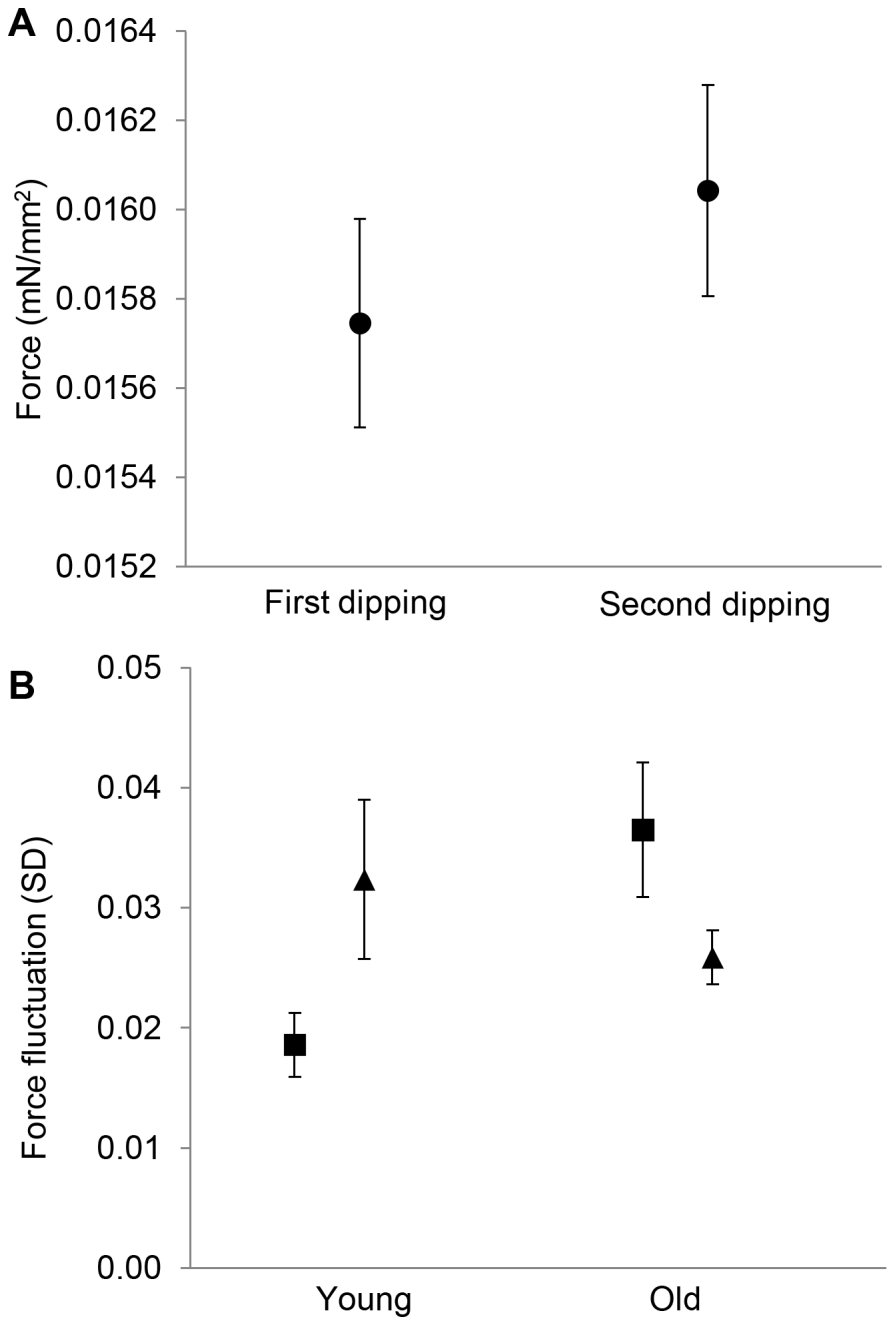
Force estimates from repeated dipping of wings. A) The force needed to dip a 1 mm^2^ of the wing area between the first and the second dipping trials (buoyant force per dipped surface area, mean ± 1se). B) Amplitude of force fluctuation during the withdrawal phase of dipping in both age groups for females (▴) and males (▪), based on means of the first and the second dippings.

During the second dipping trial, a stronger buoyant force was needed to dip a wing than during the first trial (*F*_1,25_ = 5.75, *P* = 0.024; Table 1; Fig. 5A), indicating that the wing was more water resistant during the second trial. However, trial did not have significant interactions with sex or age, nor was the three way interaction significant. When dipping trials were not taken into account in the analysis, neither sex, age, nor their interaction significantly explained variation in the buoyant force (Table 1).

**Table 1:** Results of repeated measures ANOVAs for force measurements of two repeated dippings.

Model	Source	df	F	P
**Buoyant force**	Trial	1	5.75	**0.024**
within subjects	Trial*Sex	1	2.76	0.109
effects	Trial*Age	1	0.03	0.862
	Trial*Sex*Age	1	1.67	0.208
	Error	25		
between subjects	Sex	1	0.77	0.387
effects	Age	1	1.28	0.269
	Sex*Age	1	1.09	0.306
	Error	25		
**Fluctuations**	Trial	1	3.72	0.065
within subjects	Trial*Sex	1	0.39	0.540
effects	Trial*Age	1	0.17	0.685
	Trial*Sex*Age	1	0.18	0.679
	Error	25		
between subjects	Sex	1	0.85	0.365
effects	Age	1	3.76	0.064
	Sex*Age	1	10.12	**0.004**
	Error	25		
**Pull-out** a	Trial	1	0.78	0.386
within subjects	Trial*Sex	1	0.46	0.503
effects	Trial*Age	1	3.35	0.079
	Trial*Sex*Age	1	1.68	0.207
	Error	25		
between subjects	Sex	1	1.20	0.284
effects	Age	1	3.13	0.089
	Sex*Age	1	1.74	0.200
	Error	25		

Trial is the change in a force measurement between the two repeated trials. **Buoyant force** is the force needed to dip the wing, and it includes also the force generated by water tension. **Fluctuations** indicate variation in the buoyant force during withdrawal the wing from the water (SD of the residuals of fitted curve; see Methods for further details). **Pull-out** indicates the force needed to overcome the capillary force caused by water surface tension during the withdrawal step. Statistically significant results (P<0.05) have been indicated by the bold font.

a The assumption for equality of covariance matrices were not fulfilled (Box's M = 22.79, *F*_9,1133.4_ = 2.04, *P* = 0.032).

There was marginally non-significant difference in the amplitude of buoyant force fluctuations between the first and the second dipping trial (*F*_1,25_ = 3.72, *P* = 0.065 Table 1) and non- significant two- or three-way interaction between sex and age on the amplitude of the force fluctuations between the two dipping trials. When trials were not taken into account, neither sex nor age explained variation in the amplitude of the buoyant force fluctuations during withdrawal of a wing (Table 1). Instead, sex and age had two-way interaction on the amplitude of the buoyant force fluctuations during withdrawal (*F*_1,25_ = 10.12, *P* = 0.004; Table 1; Fig. 5B). Within young individuals females had stronger force fluctuations than males (simple effects test, *P* = 0.009), but within old individuals there was no difference between the sexes (*P* = 0.115). Furthermore, older males had stronger buoyant force fluctuations during withdrawal part of dipping than younger males (*P* = 0.001), but there were no such age difference in females (*P* = 0.412).

There were no changes in the pull-out force between the first and the second dipping trials, and neither age, sex, nor their interaction significantly explained variation in pull-out force (Table 1).

### Morphological differences

*C. splendens* wings have at least two levels of structures which could be responsible for superhydrophobicity of the surface. At the microscale, wing veins were covered by spines, being heights of approximately 18 µm in the costal vein (Fig. 2A). In the costal vein, there were two rows of spines: one row oriented dorsally and another oriented ventrally. There were also setae located on these spines; a single seta could be found projecting laterally from individual spines (Fig. 2B). However, the setae have presumably a sensory function, and it remains unclear whether the setae are related to superhydrophobicity. During trials, a silverish layer could be observed over a portion of a submerged wing, especially close to wing veins, indicating that air pockets form on submerged wings. Thus, either veins themselves or their combination with spines may help to form air pockets when wings are submerged. At the nanoscale, we detected several wax rods on the wing surface: the height of individual wax rods varied from 100 to 500 nm, and varied in thickness from 30 to 45 nm. On wing veins, we observed long, filamentous wax rods (length ca. 0.5–2.0 µm; thickness 30–35 nm; Fig. 2C). Wax on females' pseudopterostigma consisted of wax rods of different heights, and wax ridges on the wing membrane (Fig. 2D).

**Figure 6:**
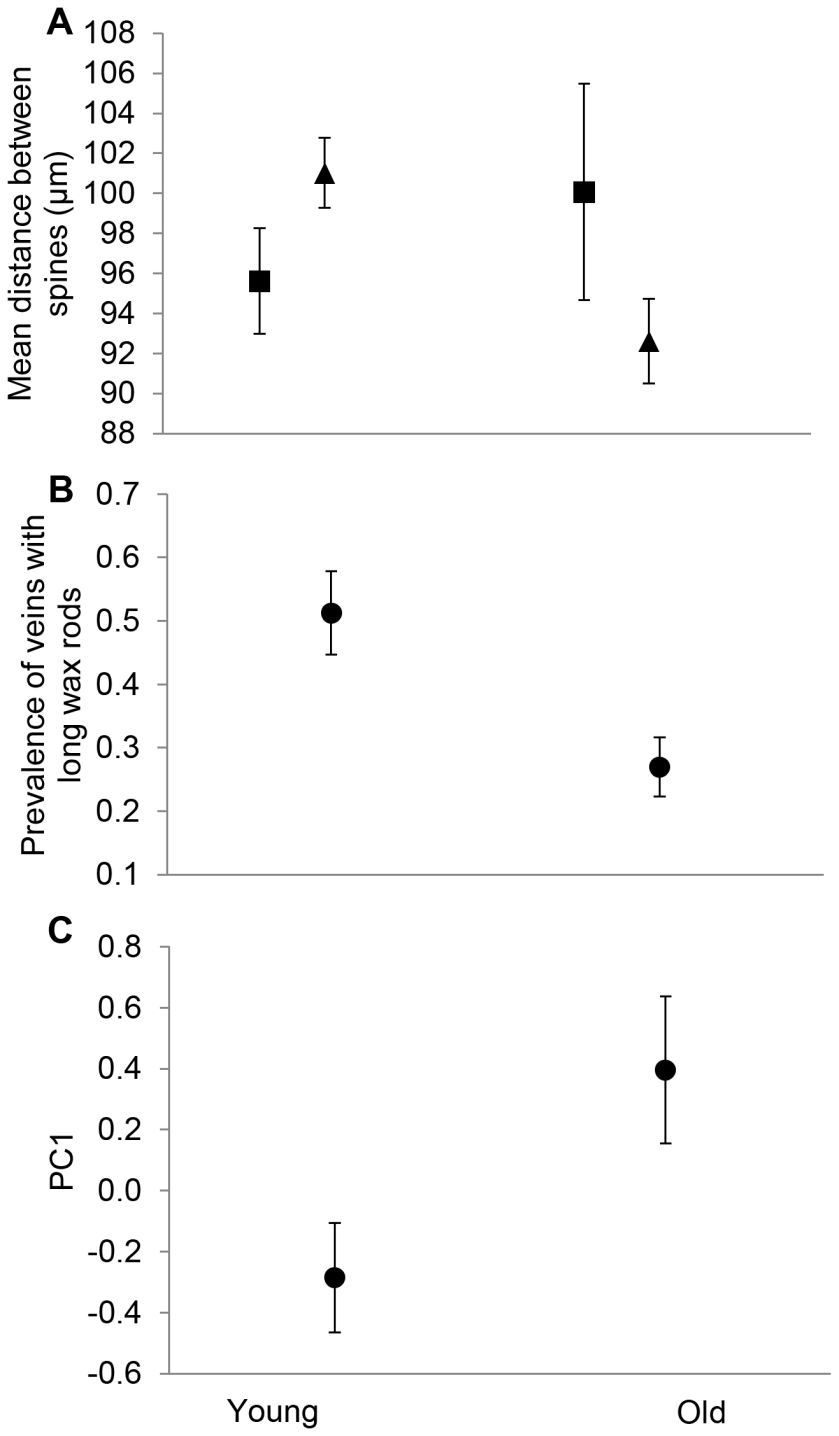
Micro- and nanoscale morphological measurements (mean ± 1se) across sex and/or age groups. A) The spine density of young and old individuals for females (▴) and males (▪). B) The prevalence of wing veins with long wax rods between young and old individuals. C) The PC1 between young and old individuals (indicating smeared and lost wax in wing veins).

The MANOVA, including all micro- and nanoscale morphological traits measured, revealed that sex had no main effects on morphological traits, but that age group did significantly explain variation in morphological traits. In addition, sex and age group had no interaction on micro- and nanostructural traits (MANOVA, Pillai's trace; Sex: *F*_5,35_ = 0.80, *P* = 0.557, Age: *F_5,35_* = 2.99, *P* = 0.024, Sex*Age: *F*_5,35_ = 1.89, *P* = 0.121; Box's test for equality of covariance matrices were not fulfilled, Box's M = 99.37, *F*_45,2254.9_ = 1.62, *P* = 0.006). A 2-way ANOVA on the spine density showed that neither sex nor age significantly explained variation in spine density, but in contrast to above analysis, there was a significant interaction between sex and age on spine density (*F*_1,25_ = 5.25, *P* = 0.027, Table 2; Fig. 6A): There were no differences in the density of spines between young and old males (simple effects test, *P* = 0.301). Instead, in young females, spines were less densely distributed than in old females (*P* = 0.027). There were no sex differences in the density of spines within young or old individuals (*P* = 0.139 and *P* = 0.093, respectively).

**Table 2:** Results from 2-way ANOVAs investigating the effect of sex and age on micro- and nanoscale morphological traits.

Model	Source	Df	F	P
**Spines** a	Sex	1	0.13	0.717
*R* ^2^ = 0.14	Age	1	0.50	0.486
	Sex*Age	1	5.25	**0.027**
	Error	39		
**Damage class**	Sex	1	1.66	0.205
*R* ^2^ = 0.09	Age	1	1.02	0.319
	Sex*Age	1	0.92	0.344
	Error	39		
**Long wax rods**	Sex	1	0.62	0.436
*R* ^2^ = 0.23	Age	1	8.50	**0.006**
	Sex*Age	1	2.12	0.154
	Error	39		
**PC1**	Sex	1	1.45	0.236
*R* ^2^ = 0.15	Age	1	4.53	**0.040**
	Sex*Age	1	0.49	0.825
	Error	39		
**PC2**	Sex	1	0.50	0.482
*R* ^2^ = 0.03	Age	1	0.18	0.676
	Sex*Age	1	0.22	0.642
	Error	39		

PC1 is positively correlated with smeared and lost wax on wing veins, and PC2 is positively correlated only with lost wax on wing veins. *R* ^2^ indicates the coefficient of determination, i.e. the part of variance which the each model explains.

a Error variances were not equal (Levene's test, *F*_3,39_ = 6.97, *P* = 0.001).

The age, but not sex, had a main effect on the prevalence of wing veins with long wax rods: the younger individuals had greater prevalence of long wax rods than the older individuals (*F*_1,25_ = 8.50, *P* = 0.006, Table 2, Fig. 6B). In addition, there was no significant sex*age interaction on the prevalence of long wax rods (Table 2). Age had a significant main effect on PC1, which means that older individuals had more smeared and lost wax on wing veins than younger individuals (*F*_1,25_ = 4.53, *P* = 0.040, Table 2, Fig. 6C). On average, 13.0±6.1% (mean ± SD) and 17.1±5.1% of the wax coverage on wing veins of young and old individuals, respectively, were lost or smeared. Sex had no main effect or interaction with age on PC1. No main effects of sex, age or their interactions were detected in damage class or PC2 (Table 2).

The area of pseudopterostigma did not differ between two female age groups (independent samples t-test, t = −0.57, df = 11, *P* = 0.577).

### Relationships between superhydrophobicity and surface morphology

**Figure 7:**
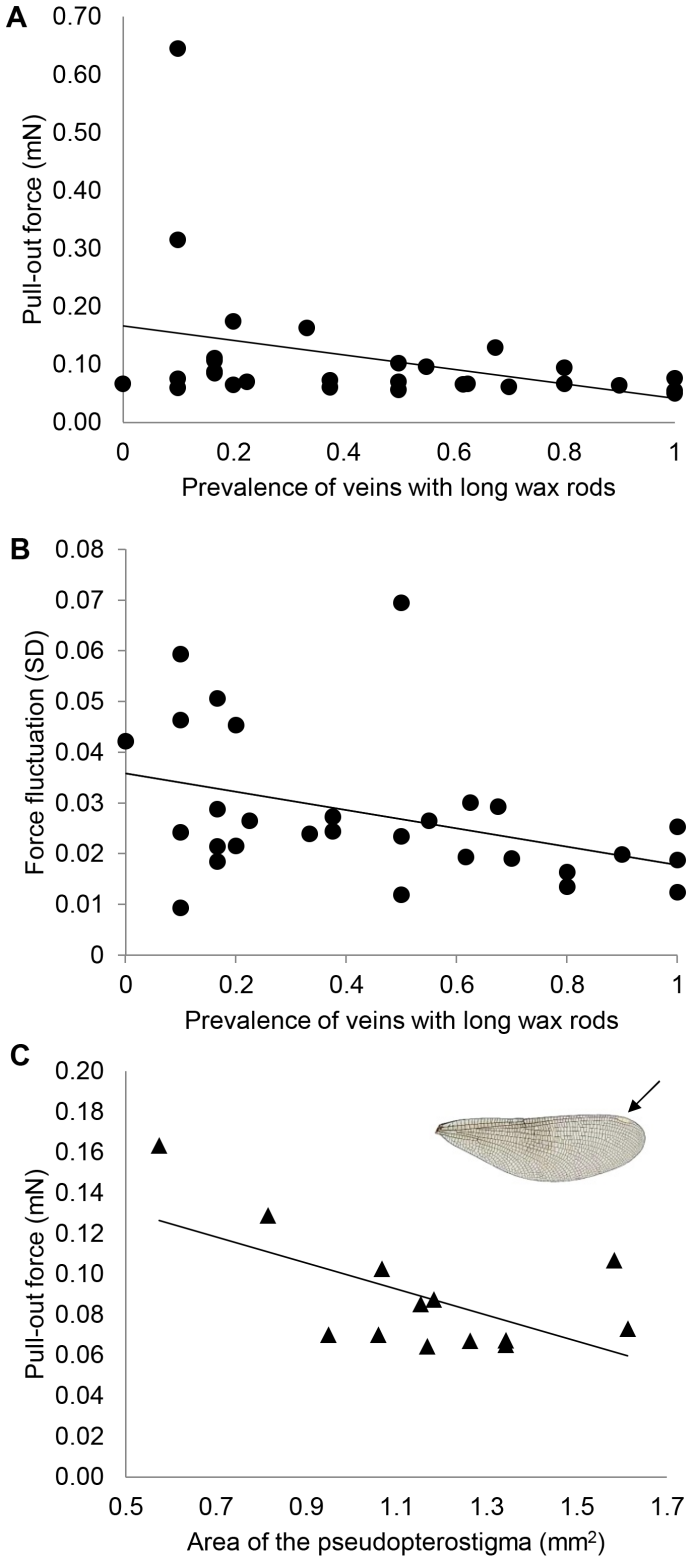
The relationships between selected morphological traits and measured forces. A) The negative correlation between the pull-out force and the prevalence of wing veins with long wax rods. B) The negative correlation between the force fluctuation during withdrawal phase of dipping (residual SDs of cubic fit regression model) and the prevalence of veins with long wax rods. C) The negative correlation between the pull-out force and the area of the pseudopterostigma in females (▴). Inset: the female wing in which the white pseudopterostigma is indicated by an arrow.

Regression analyses revealed that only two factors (the amplitude of the buoyant force fluctuations during withdrawal and the pull-out force) were significantly correlated with the prevalence of wing veins including long wax rods, having negative relationship between the two (*t* = −2.25, df = 27, *P* = 0.033, *y* = −1.49 -0.26*x*, *R* ^2^ = 0.16, and *t* = −2.34, df = 27, *P* = 0.027, *y* = −0.91−0.31*x*, *R* ^2^ = 0.17, respectively; Fig. 7A, B). The other superhydrophobicity measures (i.e. CA, buoyant force) were not related to morphological characteristics measured here, since stepwise regression did not include any of the explanatory variables in models investigating these measures (results not shown). The pull-out force and the area of the pseudopterostigma correlated negatively (Pearson correlation, *r* = −0.57, N = 13, *P* = 0.044; NS after sequential Bonferroni correction with α = 0.013; Fig. 7C). There were no correlations between other superhydrophobicity estimates and the area of the pseudopterostigma (CA: *r* = −0.35, N = 13, *P* = 0.246; buoyant force: *r* = 0.26, N = 13, *P* = 0.384; amplitude of the buoyant force fluctuations during withdrawal: *r* = −0.04, N = 13, *P* = 0.890).

## Discussion

### Hydrophobicity measurements

Contact angle (CA) measurements show that *C. splendens* have superhydrophobic wings with a mean CA of 161°, which is very close to values which have been measured from other Odonata species [10] [22] [31]. The superhydrophobicity of wing surfaces was not surprising, as several other insect species also have structures which are resistant to water (e.g. [11] [15] [16] [18] [19] [38]). Based on CA, there are no sex or age differences in the degree of superhydrophobicity. Because CA tests superhydrophobicity of a surface against water droplets, our results suggest that *C. splendens* males and females are both equally resistant to rain and splashes.

Our results from force measurements during wing submersion experiments also suggest that sex differences in the superhydrophobicity are infrequent and/or weak in *C. splendens*. The only sex related effect was for the amplitude of the buoyant force fluctuations when wings were withdrawn from water, and this effect only existed in the interaction with age: Within young individuals, females had higher amplitude of force fluctuations than males, but in old individuals, there was no difference between sexes. In addition, older males had higher amplitude of force fluctuations than younger males, but there was no such age difference in females. We suggest that high amplitudes of force fluctuations during withdrawal are indicative of wings with increased damage. Different areas of partly damaged wings may have different receding contact areas during withdrawal and thus support multiple air pockets. During the dipping trials, we observed that wings with high amplitudes of force fluctuations during withdrawals often released air as bubbles. There are two contrasting hypotheses for this sex related finding. First, and probably more likely, it may be that young females in our sample had already mated and laid eggs, and these reproductive activities had caused some damage on wing structures. For males, wing damages could accumulate more slowly. Alternatively, multiple air pockets could be beneficial for submerged *C. splendens*. If areas between the air pockets are less hydrophobic or even hydrophilic, this could stabilize the air layer on the surface of an insect and help retain air during turbulent conditions, as it does in *Salvinia* plant [21]. Thus, young females may have the possible benefits of this type of surface already when they are young. However, this is only speculation because we do not have any evidence on this kind of effect in *C. splendens* wings.

The buoyant force increased between the first and the second dippings, but there were no sex or age effects on buoyant force in *C. splendens*. Based on the increase of buoyant force, it seems that wings are more water resistant after they have been in contact with water. It may be that during dry dippings the presence of dust particles on the wings decreased buoyant force during submersion. If these particles were washed out during dry dippings, subsequent wet dippings could have become more difficult. If the observed pattern of increased buoyancy during wet submersions is truly occurring, it might be advantageous for females to lay eggs during a single submersion, because the force needed to go underwater is smaller during dry submersion than during later wet submersions. However, if reduced buoyancy also means greater drag when a female is moving underwater [8] [21], the benefit of lower buoyancy for submerging females may be marginal.

### Morphological differences

In insects, superhydrophobicity is often caused by hierarchical hydrophobic structures [10] [15] [16] [38] [39]. We observed hierarchical hydrophobic structures in the wings of *C. splendens*: At a nanoscale, wing membranes are covered with crystalline wax, and wing veins contained numerous filamentous wax rods. At a microscale, wing veins have spines. The investigation of both sex and age effects on morphology revealed that the only sex-dependent morphological trait was density of spines on wing veins. This sex-related effect, however, only appeared in an interaction with age: the spines were denser in old than young females, but males had no age differences. Within age groups, there were no differences in spine density between sexes. We propose that wing veins with dense spines have some function which increases survival of females, causing the sample of older females have dense spine cover. The spines may affect survival of females due to some other effects than just making females more hydrophobic, because hydrophobicity did not correlate with the spine density. This contradicts the idea that bristles or spines may have a function in retaining air on wings [1]. An additional benefit of spines could be the protection of veins and the wing membranes from wax damage, and thus protection of wings from loss of superhydrophobicity.

Older individuals had lower prevalence of wing veins with long wax rods, and had greater amount of smeared and lost wax coverage on wing veins (indicated by PC1) than younger individuals. Surprisingly, the observed extent of lost wax coverage seems not to have an effect on the wing superhydrophobicity, because older individuals did not have poorer water repellency in our measurements. One explanation may be that that the observed wax damages were occurring at such a small scale that they did not cause effects on superhydrophobicity. In theoretical considerations, it is known that under certain conditions, the spacing of cuticular protrusions does not necessarily affect superhydrophobicity [7]. In addition, the damaged proportion of wing veins increased with age only from 13.0% to 17.1%, which seems not to have biological significance in our study. The result of greater wax damage in older individuals contradicts our previous study (partly made with same individuals as the present study), which showed that the sample of older males had less damaged wax on abdomen [27]. In that study, we explained the obtained result by suggesting that only males which had good wax coverage were able to survive not only because of stronger water repellency of their cuticle [27] but also because wax coverage might increase pathogen resistance (see [40] and references therein) and prevention of desiccation [28] [40]. It is also probable that the importance of superhydrophobicity or micro-/nanoscale morphology on individual survival differs in different body parts. Since wings have large area compared to the rest of the damselfly body, the wax coverage of the wings should be of the greatest importance for the survival of individuals.

Because none of the morphological traits considered here showed sex differences within young individuals, our study does not answer the question of which trait caused the sex differences observed in the amplitude of the buoyant force fluctuations recorded during the withdrawal of wings from water. One possible explanation is that there are some other morphological differences between sexes, which were not measured here. In addition, surface chemistry could affect superhydrophobicity [19] [41]: if the lipid composition of the wax differs between sexes, this could explain the difference observed in the force curves. It is known from other insects that the chemical composition of surface lipids can vary between sexes [28], but to our knowledge, the lipid composition difference of wax crystals between sexes has not been studied in *Calopteryx*. Furthermore, female wings bear pseudopterostigmas (see inset of Fig. 7C), which are absent in males, and they might have an effect on water repellency.

### The relationships between morphology and superhydrophobicity

The only micro- or nanoscale morphological trait explaining superhydrophobicity of *C. splendens* was the prevalence of long wax rods in the wing veins. Both the amplitude of the buoyant force fluctuations during withdrawal and the pull-out force decreased with the increasing prevalence of long wax rods. These results suggest that air pockets are more uniform and detachment from water is easier when long wax rods are common in wing veins. Thus, it may be beneficial for an individual to have large prevalence of wing veins with long wax rods. This interpretation contradicts the possibility that large but separate air pockets could have some benefit for an individual, as we speculate earlier.

It is unclear whether the long wax rods in wing veins of *C. splendens* themselves are responsible for the observed relationships, or the existence of long wax rods correlate with the quality of wax coverage overall. Length positively correlates with superhydrophobicity in studies of artificially created nanofibers [42]. In addition, mathematical models suggest that longer protrusions may have greater role in hydrophobicity when they are in homogenous wetting regime (i.e. pockets between protrusions are also filled with water) [7]. While it is likely that wetting of *Calopteryx* wings during submersion is not homogeneous because of high CAs, wing veins may sometimes be in direct contact with water. Another possibility is that because wing veins are susceptible to mechanical damage, and long protrusions damage more easily [43], the prevalence of long wax rods is negatively correlated with overall wax coverage damage. This is supported by the negative correlation between the prevalence of long wax rods and the damage class of wax coverage (see Methods section).

Theory would not predict a strong role for long wax rods in generating superhydrophobicity as they lack structure, i.e. they are not in any certain position in relation to each other, but rather form a more or less tangle-like structure (see Fig. 2C). Less structured protrusions are generally less superhydrophobic [7]. Furthermore, long wax rods are less numerous and more patchily distributed than shorter wax rods. As a consequence, the direct effect of long, filamentous wax rods on superhydrophobicity may be limited, and the wax may have some other functions than water repellency. In insects with large quantities of filamentous wax, like in wooly alder aphid (*Prociphilus tesselatus*), the wax can have antipredatory function (see [44]). However, this function of long wax rods seems unlikely in *Calopteryx*, because filamentous wax is present in limited quantities.

The pseudopterostigma of *Calopteryx* females has previously been thought to aid in detection of females by males: females are cryptic in their colour, and a white pseudopterostigma may make female detection easier (see [45]). The area of the female pseudopterostigma and its possible role in the effect of superhydrophobicity was the only sex-related macroscopic morphological trait investigated in the present study. Our result suggests that a larger pseudopterostigma may help a female more easily detach from the water surface. Although only females bear pseudopterostigmas, there was no sex difference in the pull-out force needed to detach wings from water, suggesting that the relative benefit the pseudopterostigma provides to females in the detachment from capillary force of water may only be marginal. However, additional research is needed to verify the possibility that the pseudopterostigma provides this adaptive function.

## Conclusions

Our results demonstrate that there are some sex-related differences in the ability of *C. splendens* wings to repel water, but these differences are subtle: in contrast to young males, young females experience greater force fluctuations during withdrawal from water submersion, suggesting that they already have some damage in wax coverage. Similar superhydrophobicity of wings in both sexes could be expected in the sense that both sexes should resist rain droplets, and males can come in contact with water during territorial fights or when they try to grasp partly submerged females. With these water contacts males may have risk to be trapped by water adhesion, probably leading to strong selection for superhydrophobicity (see [15] [16] [23]). Possible sex-related adaptations are presumably too subtle to be measurable with available methods or only with short exposure to water. It is also possible that if females are selected to have stronger hydrophobicity, correlated response for the selection can be seen also in males (e.g. [46]). Additionally, we did not observe any clear micro- or nanoscale morphological differences between sexes in traits related to superhydrophobicity. At the macroscale, large pseudopterostigmas (present only on females) may facilitate emergence after submersion, but this possibility has to be verified experimentally in further studies.
